# Do Mechatronic Poles Change the Gait Technique of Nordic Walking in Patients with Ischemic Heart Disease?

**DOI:** 10.1155/2023/1135733

**Published:** 2023-06-01

**Authors:** Agnieszka Szpala, Sławomir Winiarski, Małgorzata Kołodziej, Bogdan Pietraszewski, Ryszard Jasiński, Tadeusz Niebudek, Andrzej Lejczak, Dariusz Kałka, Karolina Lorek, Krzysztof Bałchanowski, Sławomir Wudarczyk, Marek Woźniewski

**Affiliations:** ^1^Department of Biomechanics, Wroclaw University of Health and Sport Sciences, Mickiewicza 58 Street, Wrocław 51-684, Poland; ^2^Department of Human Biology, Wroclaw University of Health and Sport Sciences, Paderewskiego 35 Avenue, Wrocław 51-612, Poland; ^3^Department of Physical Culture Pedagogy, Wroclaw University of Health and Sport Sciences, Paderewskiego 35 Avenue, Wrocław 51-612, Poland; ^4^Department of Physiotherapy in Surgical Medicine and Oncology, Wroclaw University of Health and Sport Sciences, Paderewskiego 35 Avenue, Wrocław 51-612, Poland; ^5^Department of Physiotherapy in Internal Diseases, Wroclaw University of Health and Sport Sciences, Paderewskiego 35 Avenue, Wrocław 51-612, Poland; ^6^Department of Kinesiology, Wroclaw University of Health and Sport Sciences, Paderewskiego 35 Avenue, Wrocław 51-612, Poland; ^7^Department of Fundamentals of Machine Design and Mechatronics Systems, Wroclaw University of Science and Technology, Łukasiewicza 7/9 Street, Wrocław 50-371, Poland

## Abstract

The study aimed to compare the technique of normal gait with the Nordic walking (NW) gait with classical and mechatronic poles in patients with ischemic heart disease. It was assumed that equipping classical NW poles with sensors enabling biomechanical gait analysis would not cause a change in the gait pattern. The study involved 12 men suffering from ischemic heart disease (age: 66.2 ± 5.2 years, body height: 173.8 ± 6.74 cm; body mass: 87.3 ± 10.89 kg; disease duration: 12.2 ± 7.5 years). The MyoMOTION 3D inertial motion capture system (Noraxon Inc., Scottsdale, AZ, USA) was used to collect biomechanical variables of gait (spatiotemporal and kinematic parameters). The subject's task was to cover the 100 m distance with three types of gait-walking without poles (normal gait), walking with classical poles to NW, and walking with mechatronic poles from the so-called preferred velocity. Parameters were measured on the right and left sides of the body. The data were analyzed using two-way repeated measures analysis of variance with the between-subject factor “body side.” Friedman's test was used when necessary. For most kinematic parameters, with the exception of knee flexion–extension (*p* = 0.474) and shoulder flexion–extension (*p* = 0.094), significant differences were found between normal and walking with poles for both the left and right side of the body and no differences due to the type of pole. Differences between the left and right movement ranges were identified only for the ankle inversion–eversion parameter (gait without poles *p* = 0.047; gait with classical poles *p* = 0.013). In the case of spatiotemporal parameters, a reduction in the cadence step value using mechatronic poles and the stance phase using classical poles compared to normal walking was observed. There was also an increase in the values for step length and step time regardless of the type of poles, stride length, and swing phase when using classical poles and stride time when using mechatronic poles. The differences between the right and left sides of the measurement occurred when walking with both types of poles for single support (gait with classical poles *p* = 0.003; gait with mechatronic poles *p* = 0.030), stance phase (gait with classical poles *p* = 0.028; gait with mechatronic poles *p* = 0.017) and swing phase (gait with classical poles *p* = 0.028; gait with mechatronic poles *p* = 0.017). Mechatronic poles can be used in the study of the biomechanics of gait in real-time with feedback on its regularity because no statistically significant differences were found between the NW gait with classical and mechatronic poles in the studied men with ischemic heart disease.

## 1. Introduction

Gait is one of the activities that condition a person's independence. It allows obtaining the recommended level of physical activity, reducing the risk of civilization diseases. This also applies, especially to those who are ill, including those suffering from cardiovascular diseases. Gait is not only one of the basic forms of rehabilitation for these patients but also has a significant predictive value. The distance covered and the gait velocity in the walking test by patients with cardiovascular diseases are important indicators of their death risk [1–4].

According to the “muscle hypothesis,” patients with cardiovascular diseases, especially those with accompanying muscle catabolism, experience secondary changes in the function and structure of skeletal muscles, consisting of the disappearance of slow twitch fibers, secondary to the underlying disease [5, 6]. This is especially true of muscles that developed late in species development, such as the thigh's quadriceps or the calf's triceps muscle. It affects the disruption of gait, especially those phases in which these muscles show the greatest activity, i.e., stabilization of the knee joint and propulsion, showing a relationship with the reduction of exercise tolerance in patients with cardiovascular diseases. This is particularly important in the case of ankle joint function, where approximately 60% of the “driving force” is generated when walking. The weakening of the triceps muscles of the calf disrupts the function of this joint, thus reducing the “driving force” of the gait, which results, among other things, in a reduction in gait velocity. Therefore, exercises to strengthen the triceps calf muscles may be of significant importance in improving the walking ability of these patients. However, the remaining time–space parameters of walking in patients with heart disease, such as stride length, stride frequency, duty factor, stance, and swing times, did not differ statistically significantly from healthy people [7, 8].

The views on the mechanics of gait in patients with cardiovascular diseases are not clear because, in other studies, different results were obtained, showing significantly lower values of not only gait velocity but also stride length, gait cycle, and step length in these patients compared to healthy people [9]. This differentiation of the results of the gait examination in patients with cardiovascular diseases may result from examining patients at different stages of disease advancement and with different levels of activity and physical fitness. Therefore, it is necessary to continue these studies in order to obtain a reliable pattern of their gait since the inclusion of a correct gait pattern in the cardiological rehabilitation program could increase its effectiveness and improve the functional efficiency of these patients.

Physiological parameters, such as heart rate, oxygen uptake, and energy expenditure, are primarily taken into account in walking training or tests of patients with cardiovascular diseases. Among the biomechanical parameters characterizing walking in these patients, the most important are the distance and gait velocity. The literature does not use kinematic parameters and other spatiotemporal parameters as possible predictive markers of gait in patients with cardiovascular diseases. Apart from walking distance and velocity, the other parameters of walking biomechanics are usually not included in the programing of walking training. On the other hand, our previous research on walking training in patients with intermittent claudication shows that the correct gait pattern assessed with kinematic and spatiotemporal parameters significantly impacts rehabilitation effectiveness [10, 11].

Currently, the most recommended type of walking training for patients with cardiovascular diseases is Nordic walking (NW) with special poles. However, there is no developed pattern of this gait for these patients because the available literature does not include studies of kinematic and spatiotemporal parameters in this area. The articles on the use of NW in patients with cardiovascular diseases mainly concern this form of activity's physiological and health benefits [12, 13].

Mastering the correct NW gait pattern with classic poles may be difficult for older adults or people with low activity and physical fitness. In this case, classic NW poles equipped with electronic sensors (mechatronic poles), which enable real-time measurement of biomechanical parameters of gait, along with their analysis and feedback to the user, may be helpful [14]. Of fundamental importance in their reliable use in gait tests is to prove whether installing these sensors on classic NW poles will cause changes in the mechanics of gait and its pattern. There are reports on the use of such systems in the literature, but there are no kinematic and spatiotemporal studies of walking parameters with poles equipped with measuring and signaling systems.

Therefore, the study aimed to compare normal walking mechanics with NW with classic walking poles and mechatronic poles in patients with ischemic heart disease. It was assumed that equipping classic NW poles with sensors enabling biomechanical analysis of gait would not cause a change in the gait pattern.

## 2. Material and Methods

### 2.1. Material

The study was performed on a group of 12 men aged 57–71 years suffering from ischemic heart disease. The study group was characterized by poor differentiation in terms of age, height, and body weight, as indicated by the calculated values of the coefficient of variation (*V*)—7.85%, 3.88%, and 12.47%, respectively. This proves that the group was very homogeneous. Only for the duration of the disease, this coefficient was 61.47%, which proves a moderate (still acceptable) differentiation of the variable. The mean disease duration was around 12 years, with the longest duration being 31 years and the shortest 2 years. Nine patients underwent percutaneous transluminal coronary angioplasty (PTCA), two PTCA and additional coronary artery bypass grafts (CABG), and one only CABG (Table 1). There were no symptoms of heart failure in any of the patients. Before the measurements, the subjects did not know the NW gait technique.

The participants were informed about the aims and methodology used in the experiment and gave written informed consent for participation in the investigation. The experiment was approved by the local ethics committee and conducted in accordance with the Declaration of Helsinki.

### 2.2. Methods

**Figure 1:**
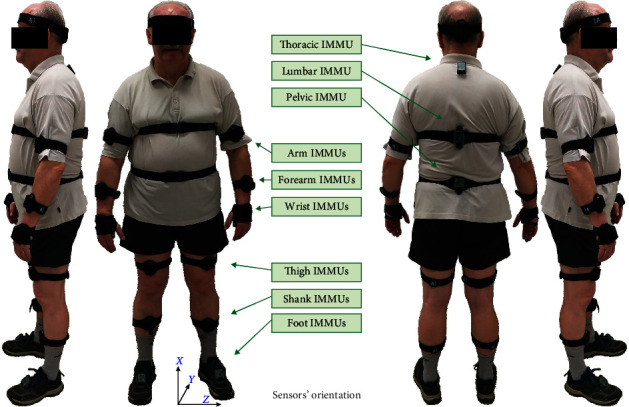
The photographic documentation of a selected participant from various perspectives, featuring 15 IMMU sensors placed on the body and the laboratory (global) frame of reference that corresponded with the reference system of each sensor. The depiction includes an observable head sensor, which was not implemented in the current investigation.

**Figure 2:**
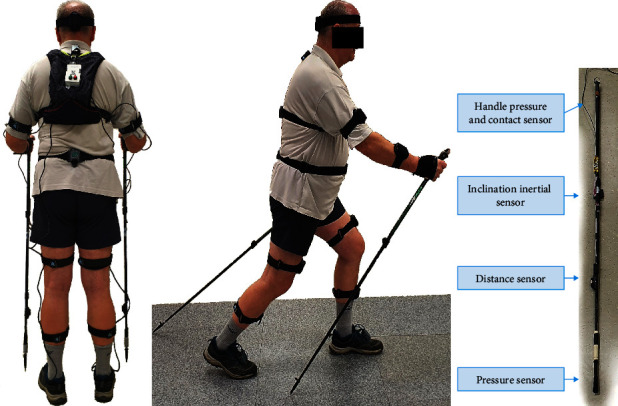
The visual depicts an individual partaking in Nordic walking while utilizing a mechatronic pole (left-hand side and center of the image) and a mechatronic pole sensorisation system (right-hand side). The depicted participant serves as a representative exemplar for the study's sample.

The MyoMOTION Research motion analysis system (Noraxon Inc., Scottsdale, Arizona, USA) collected gait kinematic variables. It is a system for the three-dimensional evaluation of motion using inertial and magnetic measurement units (IMMU) sensors. The system combines wireless data transmission and IMMU sensor technology to evaluate any motion in three-dimensional space (e.g., changes in angles between segments and linear acceleration). Each sensor included an accelerometer, gyroscope, and magnetometer for measuring the earth's magnetic field. The IMMU sensors were placed on the subject's body according to a model compatible with Noraxon MR3 software, which enabled both data recording and comprehensive analysis (Figures 1 and 2). The sensors sampling rate used in this study was 200 Hz. A total of 15 sensors were attached, three for each upper and lower limb (right/left) and three for the spinal region (on the spinous process of the 7th cervical and 7th thoracic vertebrae and in the sacral region). Sensors were attached with elastic straps and self-adhesive tape symmetrically so that the *y*-coordinate corresponded to the frontal horizontal axis and the *z*-coordinate to the sagittal horizontal axis of the segment (Figure 1). The positive vertical *x*-coordinate on the sensor label corresponded to a superior orientation for the trunk, head, and pelvis (Figure 1). The positive *x*-coordinate corresponded to a proximal orientation for the limb segment sensors. The *x*-coordinate was directed distally (to the toes) for the foot sensor. The sensors were placed according to the MyoMOTION protocol described in the manual. Each participant was checked at the beginning of the measurement, and the system was calibrated according to the factory's recommendations.

The subject's task was to walk a 100 m distance in a straight line using three types of gait: walking without poles (normal), walking with classical NW poles, and walking with mechatronic poles (Figure 2). The basic step with poles to NW took place after a short instruction from the instructor. The walk occurred on a pitch with an artificial surface at the so-called natural (preferred) velocity. Two trials were performed for each type of gait, which allowed to obtain an average of 70 complete gait cycles per trial.

Mechatronic NW poles, equipped with a system for measuring, diagnosing, and monitoring the patient's gait, were developed as part of the Wroclaw University of Science and Technology research project in cooperation with the Wroclaw University of Health and Sport Sciences. The design of the mechatronic poles has been extensively described in prior articles [15–17]. In summary, the conventional NW pole has a single inertial sensor consisting of a three-axis gyroscope, accelerometer, and magnetometer (Waveshare IMU 10DoF). The foot of the pole has a pressure sensor that measures the force along the pole's axis, and the handle contains another pressure sensor (Tekscan FlexiForce A201). Additionally, the pole has optical distance sensors (Sharp GP2Y0D340K sensor). The attached mechatronic components have increased the weight of the standard NW pole by 0.1 kg. The tests conducted on signals from the inertial sensors, pressure sensors, and distance sensors have confirmed sufficient accuracy for the study of gait biomechanics [17–19].

### 2.3. Data Registration and Signal Processing

The following parameters were recorded separately for the right (RT) lower/upper limb and for the left (LT) lower/upper limb:

1. Spatiotemporal parameters related to the step cycle (%): stance phase duration, load response duration, single support duration, preswing duration, swing phase duration, and double stance duration. Step time (ms), stride time (ms), stride length (cm), velocity (m/s), and step frequency (cadence) (step/min) were also recorded.
2. Range of motion (ROM) expressed in angular degrees (deg) at the following joints: shoulder (flexion–extension, abduction–adduction, internal–external rotation), elbow (flexion–extension), wrist (flexion–extension, radial–ulnar), hip (flexion–extension, abduction–adduction, internal–external rotation), knee (flexion–extension), ankle (dorsiflexion–plantarflexion, inversion–eversion, abduction–adduction).

The raw IMMU data (from the accelerometer, gyroscope, and magnetometer) was captured for customized sensor fusion and analysis using the MyoResearch 3 dedicated software. This procedure included the following steps: choosing a predefined measurement set-up, checking the signals from the sensors, static calibration, registration of the movement task, checking for movement artifacts, semiautomated identification of movement cycles and phases, and storing the records in the project folder for further analysis. Calibration of the IMMU sensor for body position was performed before each measurement. The standing position with arms parallel along the torso was used to determine the value of the 0° angle as a calibration posture. The maximum sampling rate for a given sensor was 100 Hz per sensor for the 16-sensor set (excluding the head sensor). We used system-build fusion algorithms and Kalman filtering (digital bandpass FIR filter). This approach allowed direct access to all unprocessed IMMU sensor data for further computations. The MyoMOTION system is outfitted with virtual footswitches that rely on data from assigned foot sensors' gyroscopes and accelerometers to discern instances of foot contact. These footswitches enable users to observe a subject's steps visually and offer the added benefit of facilitating the use of the MyoMOTION Gait Foot Switch Report. This report employs the stance and swing phases to ascertain spatial gait parameters and depicts averaged kinematic angle curves using footswitch data to determine temporal intervals.

All analyses were performed using MyoResearch MR3 (Master Edition) software (Noraxon Inc., Scottsdale, Arizona, USA), including a preinstalled MyoMOTION gait report for IMMU-based 3D kinematic records, allowing for computation of standard amplitude analysis with mean, min/max value separately for upper and lower limb movements and each subject.

### 2.4. Statistical Analysis

The values of all measurements are presented as mean ± standard deviation (*M* ± SD). The normality of the distribution of the variables was checked with the Shapiro–Wilk test. Differences between the three types of gait were tested by repeated measures analysis of variance (repeated measures ANOVA) with the body side (right vs. left) as a grouping factor. Homogeneity of variance was verified by Box's *M* test, sphericity by the Mauchly test, Greenhouse–Geisser correction, and multivariate tests with Wilks' lambda were applied where necessary. Tukey's test (also known as the honest significant difference (HSD) test) was used for multiple comparisons. Friedman test and Dunn–Bonferroni post hoc test were used when ANOVA assumptions were violated. Both Tukey and Dunn–Bonferroni post hoc tests control the Type I error independently.

All analyses were performed using Statistica 13.3.0 (TIBCO Software Inc., Palo Alto, CA). The statistical significance of the results was accepted at *p* < 0.05.

## 3. Results

**Figure 3:**
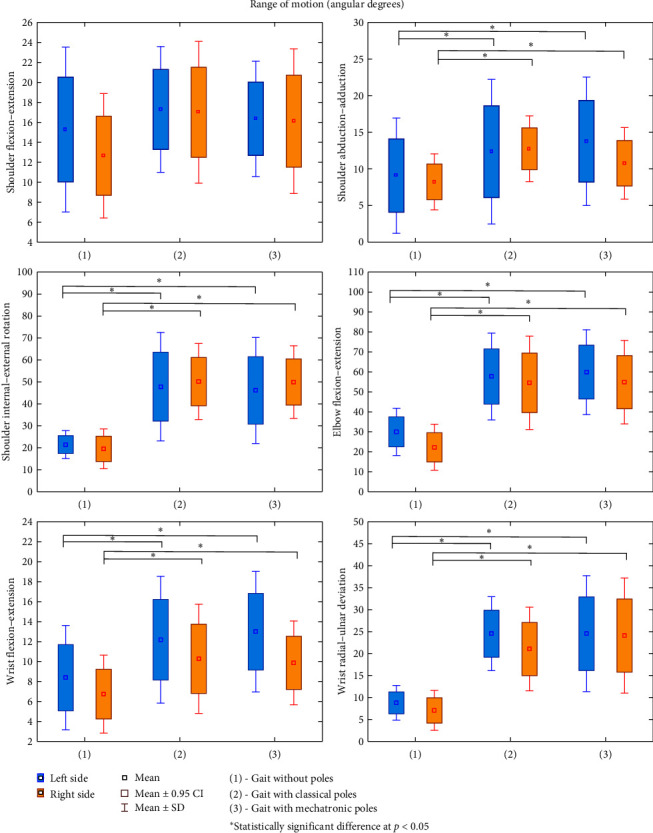
Differences between the kinematic parameters of gait: normal (without poles) (1) vs. “Classical poles” (2) vs. “Mechatronic poles” (3) for upper limbs in patients with ischemic heart disease (*n* = 12).

**Figure 4:**
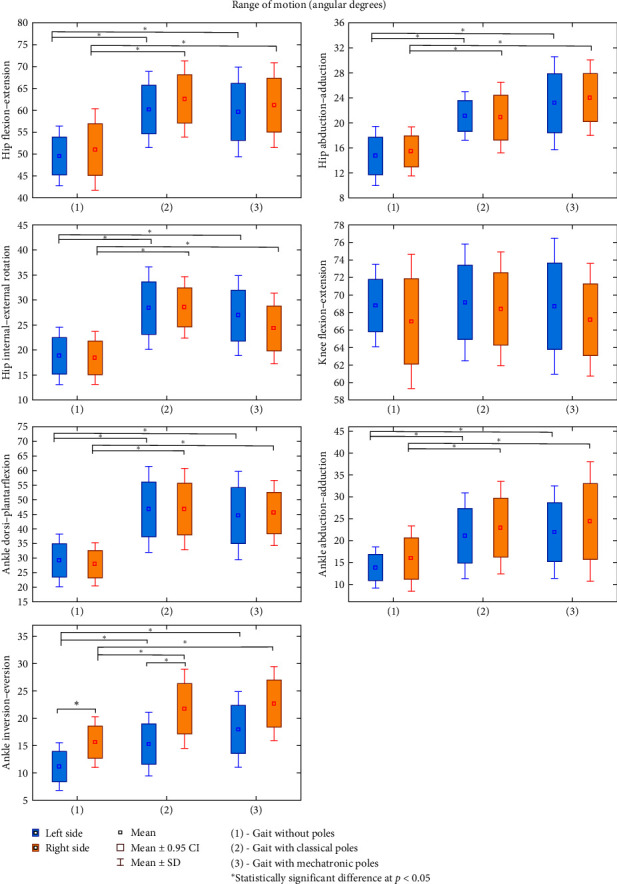
Differences between the kinematic parameters of gait: normal (without poles) (1) vs. “Classical poles” (2) vs. “Mechatronic poles” (3) for lower limbs in patients with ischemic heart disease (*n* = 12).

**Figure 5:**
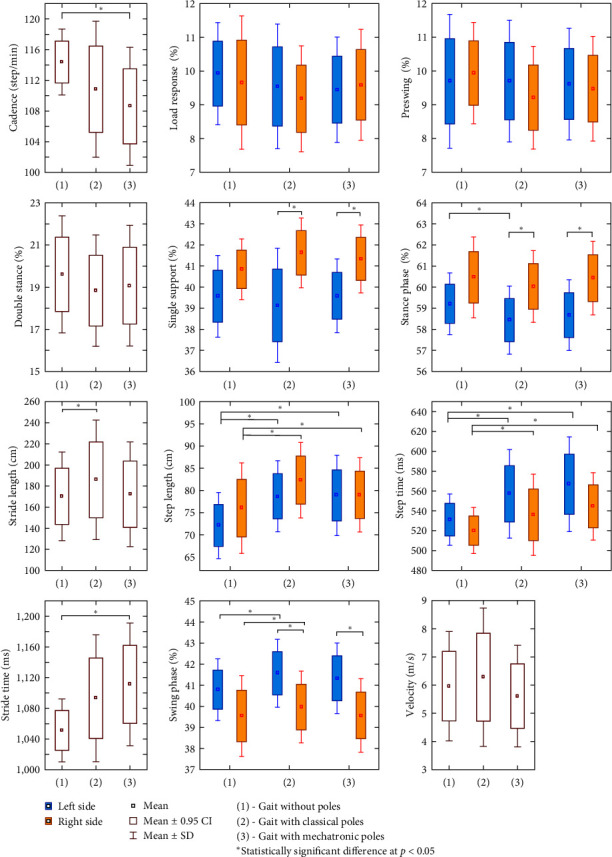
Differences between spatiotemporal parameters of gait: normal (without poles), (1) vs. “Classical poles” (2) vs. “Mechatronic poles” (3) in patients with ischemic heart disease (*n* = 12).

The results of all analyzed kinematic parameters of gait measured in three measurements: without poles (1), with classical poles (2), and with mechatronic poles (3) in a group of 12 patients are presented as mean ± standard deviation (*M* ± SD) in Figures 3–5 (and Table S1 and S2 included in the Supplementary Materials). For most of the parameters, with the exception of shoulder flexion–extension (Figure 3) and knee flexion–extension (Figure 4), significant differences were found between normal and walking with poles for both the left and right side of the measurement, and no differences due to the type of pole. For all kinematic parameters of the gait for which differences between the measurements were confirmed, the values for walking with both classical and mechatronic poles were significantly higher than those recorded during normal walking (without sticks). The differences between the measurements for the left and right sides were identified for the ankle inversion–eversion parameter (gait without poles *p* = 0.047; gait with classical poles *p* = 0.013). When walking without poles and with classical poles, the ankle inversion–eversion values were higher for the right limb than for the left limb (Figure 4). No significant differences between the right and left sides were found for the remaining ROM, regardless of the use of poles and their types (*p* > 0.05).

In the case of the spatiotemporal parameters of gait and the kinematic parameters, all the confirmed differences concerned changes caused by the use of poles while walking and not their type (Figure 5). Compared to normal gait, a reduction in the cadence step value was observed using mechatronic poles and the stance phase using classical poles. An increase in the value when using poles was found for step length and step time regardless of the type of poles, stride length, and swing phase when using classical poles and stride time when using mechatronic poles. The differences between the right and left sides of the measurement occurred when walking with both types of poles for single support (gait with classical poles *p* = 0.003; gait with mechatronic poles *p* = 0.030), stance phase (gait with classical poles *p* = 0.028; gait with mechatronic poles *p* = 0.017), and swing phase (gait with classical poles *p* = 0.028; gait with mechatronic poles *p* = 0.017).

## 4. Discussion

The analysis of the obtained results showed statistically significant differences between the studied types of gait for 11 out of 13 measured kinematic parameters and 7 out of 12 spatiotemporal parameters. As in the results of other authors, greater ROMs in the joints of the lower and upper limbs during NW walking compared to normal walking were confirmed, which indicates their greater involvement when walking with poles. Increasing the hip joint flexion range during NW gait increased the share of the swing phase without changing the share of the single and double support phase during walking with poles compared to normal walking. This resulted in a significant increase in stride length and shortening of the cadence, which was to be expected as a natural consequence of a swing phase, but without an increase in gait velocity [12, 13].

Higher values of the range of movements in the frontal and transverse planes in the joints of the lower extremities during NW gait with poles may suggest an abnormal gait pattern in the examined patients with ischemic heart disease. People with the highest level of NW gait technique show only higher values of the ROM in the sagittal plane when walking with poles compared to normal gait.

The manifestation of an incorrect NW gait technique in patients with ischemic heart disease could also be significantly greater extensions in the wrist joint, i.e., movements in the sagittal plane with the position of the hand holding the pole during this gait. On the other hand, the movement in this joint should take place only in the frontal plane, so with the position of the hand holding the pole during NW gait. Larger ranges should concern radial and ulnar range in the wrist joint, which was confirmed in our research (*p* < 0.001) because their values were almost three times greater with NW gait compared to normal walking.

There was no significant reduction in load response (*p* = 0.246) during NW walking in patients with ischemic heart disease compared to normal walking. This was to be expected as a result of partial load on the lower limbs supported by the upper limbs supported by poles and which does not confirm the observation of other authors who found significantly lower limb load during NW gait [20]. However, the results of our research are consistent with the authors' views, who showed no significant differences in the hemodynamic response during NW gait compared to normal walking, considering walking with poles as a safe form of physical activity for patients with cardiovascular diseases [13, 21]. Perhaps patients with cardiovascular diseases, subconsciously limiting the participation of upper limbs during NW gait, do not burden to the same extent as healthy people with extensive experience and advancement in walking with poles.

It is important because exercises involving the upper body muscles are associated with a proportionally greater load on the heart muscle, increasing the load on the cardiovascular system, which in the case of patients with cardiovascular diseases, may increase the risk of cardiac events. Therefore, a greater share of the upper limbs in exercises during the rehabilitation of patients with cardiovascular diseases indicates performing an exercise test using an ergometer for the upper limbs before starting training. This should also apply to the NW gait, which causes greater involvement of the upper limbs than the normal gait (without poles), which is manifested, among other things, by higher values of the ROM in the joints of these limbs.

The differences found in some parameters between the right and left sides of the body during NW gait could result from the subconscious protection and sparing of the left half of the body, which is quite characteristic of patients with ischemic heart disease. One of the symptoms is pain behind the breastbone radiating to the left shoulder, which is aggravated by exercise. This can produce a reflex analgesic reaction of less involvement of the left upper and lower limbs in walking, which can become permanent and become a habit, affecting the asymmetric gait pattern with poles. This could result in longer single support on the right limb with a simultaneous extension of the left limb swing phase during NW gait with both classical and mechatronic poles. These asymmetries did not occur during normal walking (without poles), probably because the share of limbs in this type of gait is significantly lower than during NW gait. This causes much greater involvement of the limbs and, thus, greater physical effort, which could be associated with patients suffering from ischemic heart disease with pain. One should pay attention to learning NW gait and, later, during training because such asymmetries that do not occur in professional walking with poles may cause an incorrect gait pattern. They can affect not only the effectiveness of rehabilitation but also increase the risk of developing overload changes in the locomotor system.

The lack of significant differences in spatiotemporal and kinematic parameters of NW gait with classical poles and mechatronic poles in men suffering from ischemic heart disease indicates that additional sensors mounted on poles did not constitute a factor that would change the gait technique and pattern depending on the type of poles. Due to the lack of significant differences in walking with classical and mechatronic NW poles (after taking into account the comments on the correct technique of NW walking and, in particular, eliminating excessive movements in the frontal and transverse planes and asymmetry), mechatronic poles could constitute, in real-time, a reliable method of analysis and information on the biomechanics of NW gait in patients with ischemic heart disease, significantly supporting and increasing the effectiveness of cardiac rehabilitation.

## 5. Conclusion

Walking with NW poles increased the proportion of lower and upper limbs in patients with ischemic heart disease compared to the normal gait (without poles). The NW gait pattern with poles in these patients was characterized by an increased share of the swing phase, a longer stride length, and a shorter cadence without changing the single and double support phase and gait velocity. The ROM in the joints of the lower limbs in the frontal and transverse planes and the extension of the wrist joint during NW gait in patients with ischemic heart disease could be a symptom of an incorrect technique of this gait, with larger values observed. Mechatronic poles can be used in real-time with feedback on the correctness of gait biomechanics, as no statistically significant differences were found between the NW gait with classical and mechatronic poles in the studied group with ischemic heart disease.

## Figures and Tables

**Table 1:** Characteristics of the study group.

Subject	Age (years)	Body height (m)	Body mass (kg)	Disease duration (years)	PTCA/CABG
1	64	1.82	104	31	−/−
2	66	1.74	74	12	+/+
3	74	1.84	81	17	+/−
4	68	1.80	98	2	+/−
5	69	1.67	77	7	+/−
6	57	1.75	79	9	+/−
7	69	1.65	89	9	−/+
8	57	1.71	78	5	+/−
9	71	1.61	78	8	+/−
10	67	1.76	88	11	+/−
11	61	1.79	108	14	+/+
12	71	1.71	93	21	−/−
Mean ± SD	66.2 ± 5.2	1.738 ± 6.74	87.3 ± 10.89	12.2 ± 7.5	Total 9/3

SD, standard deviation; PTCA, percutaneous transluminal coronary angioplasty; CABG, coronary artery bypass grafting.

## Data Availability

The data used to support the findings of this study are available from the corresponding author upon request.

## Additional Points

*Limitations*. The research was performed in a relatively small group, which may increase the risk of a statistical error. The analysis of the obtained results did not take into account the degree of disease advancement as well as the activity and physical fitness of the studied men, which could have had an impact on the parameters tested. In the future, research should be carried out on a larger group of patients with ischemic heart disease, taking into account the disease advancement stage as well as their activity and physical fitness as independent variables. Similar tests should also be performed in the group of women because sex may also differentiate the gait pattern of patients with ischemic heart disease.

## Supplementary Materials

Supplementary Materials Table S1: Differences between the kinematic parameters of gait: normal (without poles) (1) vs. “Classical poles” (2) vs. “Mechatronic poles” (3). Table S2: Differences between spatiotemporal parameters of gait: normal (without poles), (1) vs. “Classical poles” (2) vs. “Mechatronic poles” (3) in patients with ischemic heart disease.

## References

[1] Roy M., Grattard V., Dinet C., Soares A. V., Decavel P., Sagawa Y. J. (2020). Nordic walking influence on biomechanical parameters: a systematic review. *European Journal of Physical and Rehabilitation Medicine*.

[2] Dodson J. A., Arnold S. V., Gosch K. L. (2016). Slow gait speed and risk of mortality or hospital readmission after myocardial infarction in the translational research investigating underlying disparities in recovery from acute myocardial infarction: patients’ health status registry. *Journal of the American Geriatrics Society*.

[3] Flint K., Kennedy K., Arnold S. V., Dodson J. A., Cresci S., Alexander K. P. (2018). Slow gait speed and cardiac rehabilitation participation in older adults after acute myocardial infarction. *Journal of the American Heart Association*.

[4] Avram R. L., Nechita A. C., Popescu M. N. (2022). Functional tests in patients with ischemic heart disease. *Journal of Medicine and Life*.

[5] Coats A. J. S., Clark A. L., Piepoli M., Volterrani M., Poole-Wilson P. A. (1994). Symptoms and quality of life in heart failure: the muscle hypothesis. *Heart*.

[6] Clark A. L., Poole-Wilson P. A., Coats A. J. S. (1996). Exercise limitation in chronic heart failure: central role of the periphery. *Journal of the American College of Cardiology*.

[7] Panizzolo F. A., Maiorana A. J., Naylor L. H. (2015). Is the soleus a sentinel muscle for impaired aerobic capacity in heart failure?. *Medicine & Science in Sports & Exercise*.

[8] Panizzolo F. A., Maiorana A. J., Naylor L. H. (2014). Gait analysis in chronic heart failure: the calf as a locus of impaired walking capacity. *Journal of Biomechanics*.

[9] Ozcan E. B., Saglam M., Vardar-Yagli N. (2022). Impaired balance and gait characteristics in patients with chronic heart failure. *Heart, Lung and Circulation*.

[10] Pietraszewski B., Woźniewski M., Jasiński R., Struzik A., Szuba A. (2019). Changes in gait variables in patients with intermittent claudication. *BioMed Research International*.

[11] Dziubek W., Stefańska M., Bulińska K. (2020). Effects of physical rehabilitation on spatiotemporal gait parameters and ground reaction forces of patients with intermittent claudication. *Journal of Clinical Medicine*.

[12] Cugusi L., Manca A., Yeo T. J., Bassareo P. P., Mercuro G., Kaski J. C. (2017). Nordic walking for individuals with cardiovascular disease: a systematic review and meta-analysis of randomized controlled trials. *European Journal of Preventive Cardiology*.

[13] Girold S., Rousseau J., Le Gal M., Coudeyre E., Le Henaff J. (2017). Nordic walking versus walking without poles for rehabilitation with cardiovascular disease: randomized controlled trial. *Annals of Physical and Rehabilitation Medicine*.

[14] Mocera F., Aquilino G., Somà A. (2018). Nordic walking performance analysis with an integrated monitoring system. *Sensors*.

[15] Szrek J., Muraszkowski A., Bałchanowski J., Rusiński E., Pietrusiak D. (2019). Force measurement module for mechatronic Nordic walking poles. *Proceedings of the 14th International Scientific Conference: Computer Aided Engineering*.

[16] Wudarczyk S., Szrek J., Bałchanowski J. Research on the mechatronic gait monitoring system with nordic walking poles.

[17] Wudarczyk S., Szrek J., Bałchanowski J. Experimental research on mechatronic nordic walking poles.

[18] Szpala A., Kołodziej M., Struzik A. (2022). Selected spatiotemporal and joint angle parameters in normal gait and nordic walking with classical and mechatronic poles in aspects of sex differences. *BioMed Research International*.

[19] Szpala A., Winiarski S., Kołodziej M. (2023). No influence of mechatronic poles on the movement pattern of professional nordic walkers. *International Journal of Environmental Research and Public Health*.

[20] Park S. K., Yang D. J., Kang Y. H., Kim J. H., Uhm Y. H., Lee Y. S. (2015). Effects of Nordic walking and walking on spatiotemporal gait parameters and ground reaction force. *Journal of Physical Therapy Science*.

[21] Knobloch K. (2009). No difference in the hemodynamic response to Nordic pole walking vs. conventional brisk walking—a randomized exercise field test using the ultrasonic cardiac output monitor (USCOM). *International Journal of Cardiology*.

