# Supplementary material for: Do Mechatronic Poles Change the Gait Technique of Nordic Walking in Patients with Ischemic Heart Disease?
Source: Appl Bionics Biomech. 2023 Jun 1;2023:1135733. doi: 10.1155/2023/1135733 (PMC10250095; doi:10.1155/2023/1135733)
Supplement: Supplementary Materials — Table S1: Differences between the kinematic parameters of gait: normal (without poles) (1) vs. “Classical poles” (2) vs. “Mechatronic poles” (3). Table S2: Differences between spatiotemporal parameters of gait: normal (without poles), (1) vs. “Classical poles” (2) vs. “Mechatronic poles” (3) in patients with ischemic heart disease. [file 1135733.f1.docx]

### **Table S1.** Differences between the kinematic parameters of gait: normal (without poles) (1) vs. "Classical poles" (2) vs. "Mechatronic poles" (3) in patients with ischemic heart disease (n = 12).

| **ROM**  **(deg)** |  | **Gait without poles**  **(1)**  **M±SD** | **Gait with classical poles**  **(2)**  **M±SD** | **Gait with mechatronic poles**  **(3)**  **M±SD** | **p-value for**  **the NOVA or Friedman test** | **Sign. difference (1)-(2)-(3)**  **(according to post-hoc test)** |
| --- | --- | --- | --- | --- | --- | --- |
| **Upper limb movements** |  |  |  |  |  |  |
| Shoulder Flexion–Extension | LT | 15.28 ± 8.25 | 17.29 ± 6.30 | 16.35 ± 5.77 | *0.094* |  |
|  | RT | 12.66 ± 6.23 | 17.01 ± 7.10 | 16.11 ± 7.25 |  |  |
| Shoulder Ab.–Adduction | LT | 9.08 ± 7.88 | 12.35 ± 9.88 | 13.77 ± 8.76 | *0.011* | (1)-(2) |
|  | RT | 8.24 ± 3.83 | 12.74 ± 4.50 | 10.76 ± 4.89 |  | (1)-(3) |
| Shoulder Int.–Ext. Rotation | LT | 21.47 ± 6.41 | 47.83 ± 24.68 | 46.11 ± 24.18 | *<0.001* | (1)-(2) |
|  | RT | 19.53 ± 9.09 | 50.16 ± 17.33 | 49.93 ± 16.54 |  | (1)-(3) |
| Elbow Flexion–Extension | LT | 29.97 ± 11.83 | 57.70 ± 21.76 | 59.91 ± 21.24 | *<0.001* | (1)-(2) |
|  | RT | 22.26 ± 11.49 | 54.53 ± 23.40 | 54.87 ± 20.91 |  | (1)-(3) |
| Wrist Flexion–Extension | LT | 8.39 ± 5.23 | 12.19 ± 6.36 | 12.99 ± 6.04 | *0.021* | (1)-(2) |
|  | RT | 6.74 ± 3.91 | 10.28 ± 5.47 | 9.86 ± 4.20 |  | (1)-(3) |
| Wrist Radial–Ulnar Deviation | LT | 8.79 ± 3.94 | 24.52 ± 8.43 | 24.53 ± 13.19 | *<0.001* | (1)-(2) |
|  | RT | 7.05 ± 4.54 | 21.03 ± 9.52 | 24.10 ± 13.09 |  | (1)-(3) |
| **Lower Limb movements** |  |  |  |  |  |  |
| Hip Flexion–Extension | LT | 49.56 ± 6.82 | 60.19 ± 8.70 | 59.63 ± 10.25 | <0.001 | (1)-(2) |
|  | RT | 51.04 ± 9.30 | 62.61 ± 8.71 | 61.17 ± 9.70 |  | (1)-(3) |
| Hip Abduction–Adduction | LT | 14.70 ± 4.73 | 21.10 ± 3.88 | 23.13 ± 7.44 | <0.001 | (1)-(2) |
|  | RT | 15.43 ± 3.92 | 20.84 ± 5.65 | 24.04 ± 6.04 |  | (1)-(3) |
| Hip Int.–External Rotation | LT | 18.82 ± 5.74 | 28.37 ± 8.25 | 26.88 ± 7.99 | <0.001 | (1)-(2) |
|  | RT | 18.42 ± 5.28 | 28.52 ± 6.13 | 24.31 ± 7.06 |  | (1)-(3) |
| Knee Flexion–Extension | LT | 68.80 ± 4.71 | 69.16 ± 6.67 | 68.72 ± 7.77 | 0.474 |  |
|  | RT | 98.00 ± 7.68 | 68.42 ± 6.50 | 67.17 ± 6.44 |  |  |
| Ankle Dorsi–Plantarflexion | LT | 29.20 ± 9.03 | 46.68 ± 14.76 | 44.58 ± 15.14 | <0.001 | (1)-(2) |
|  | RT | 27.87 ± 7.39 | 46.81 ± 13.98 | 45.45 ± 11.17 |  | (1)-(3) |
| Ankle Abduction–Adduction | LT | 13.86 ± 4.71 | 21.10 ± 9.79 | 21.92 ± 10.58 | *<0.001* | (1)-(2) |
|  | RT | 15.89 ± 7.46 | 22.95 ± 10.58 | 24.36 ± 13.64 |  | (1)-(3) |
| Ankle Inversion–Eversion | LT | **11.16 ± 4.36** | **15.28 ± 5.82** | 17.97 ± 6.91 | <0.001 | (1)-(2) |
|  | RT | **15.65 ± 4.64** | **21.74 ± 7.24** | 22.70 ± 6.76 |  | (1)-(3) |

### Legend: The results of Friedman's test are in italics; significant differences between LT and RT are marked in bold; M - Mean; SD - standard deviation; LT, RT - left and right sides, respectively.

### **Table S2.** Differences between spatio-temporal parameters of gait: normal (without poles), (1) vs "Classical poles" (2) vs "Mechatronic poles" (3) in patients with ischemic heart disease (n = 12).

| Parameters: |  | **Gait without poles**  **(1)**  **M±SD** | **Gait with classical poles**  **(2)**  **M±SD** | **Gait with mechatronic poles**  **(3)**  **M±SD** | **p-value for**  **the NOVA or Friedman test** | **Sign. difference (1)-(2)-(3)**  **(according to post-hoc test)** |
| --- | --- | --- | --- | --- | --- | --- |
| Cadence (step/min) |  | 114.39 ± 4.3 | 110.84 ± 8.87 | 108.6 ± 7.7 | 0.036 | (1)-(3) |
| Double stance (%) |  | 19.61 ± 2.78 | 18.83 ± 2.64 | 19.07 ± 2.86 | 0.325 |  |
| Load response (%) | LT | 9.93 ± 1.51 | 9.55 ± 1.85 | 9.45 ± 1.56 | 0.246 |  |
|  | RT | 9.66 ± 1.98 | 9.18 ± 1.57 | 9.59 ± 1.64 |  |  |
| Pre-swing (%) | LT | 9.69 ± 1.99 | 9.7 ± 1.8 | 9.61 ± 1.66 | *0.327* |  |
|  | RT | 9.94 ± 1.5 | 9.21 ± 1.52 | 9.48 ± 1.55 |  |  |
| Single support (%) | LT | 39.57 ± 1.94 | **39.13 ± 2.71** | **39.59 ± 1.75** | *0.746* |  |
|  | RT | 40.85 ± 1.44 | **41.62 ± 1.66** | **41.34 ± 1.61** |  |  |
| Stance phase (%) | LT | 59.21 ± 1.46 | **58.43 ± 1.61** | **58.67 ± 1.68** | 0.016 | (1)-(2) |
|  | RT | 60.46 ± 1.92 | **60.03 ± 1.7** | **60.43 ± 1.74** |  |  |
| Step length (cm) | LT | 72.1 ± 7.43 | 78.69 ± 8.01 | 78.89 ± 9.05 | *0.002* | (1)-(2) |
|  | RT | 76.02 ± 10.21 | 82.32 ± 8.53 | 79.00 ± 8.37 |  | (1)-(3) |
| Step time (ms) | LT | 531.13 ± 25.85 | 557.1 ± 44.62 | 566.74 ± 47.79 | 0.002 | (1)-(2) |
|  | RT | 520.11 ± 23.24 | 535.95 ± 40.91 | 544.5 ± 34.01 |  | (1)-(3) |
| Stride length (cm) |  | 172.26 ± 49.63 | 185.92 ± 56.66 | 170.27 ± 42.01 | *0.018* | (1)-(2) |
| Stride time (ms) |  | 1051.3 ± 41.1 | 1093.1 ± 82.7 | 1111.3 ± 80.0 | 0.021 | (1)-(3) |
| Swing phase (%) | LT | 40.79 ± 1.46 | **41.57 ± 1.61** | **41.33 ± 1.68** | 0.016 | (1)-(2) |
|  | RT | 39.54 ± 1.92 | **39.97 ± 1.70** | **39.57 ± 1.74** |  |  |
| Velocity (m/s) |  | 1.65 ± 0.54 | 1.74 ± 0.68 | 1.56 ± 0.5 | *0.353* |  |

### Legend: The results of Friedman's test are in italics; significant differences between LT and RT are marked in bold; M - Mean; SD - standard deviation; LT, RT - left and right sides, respectively.
